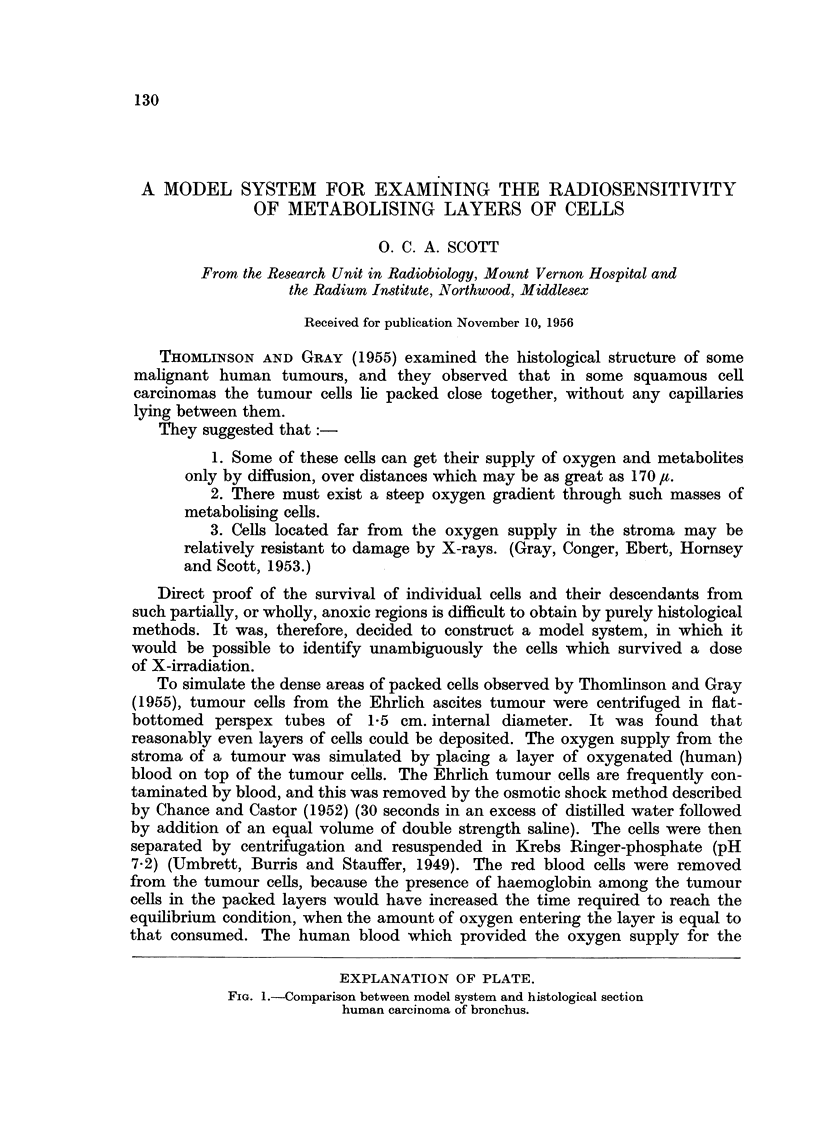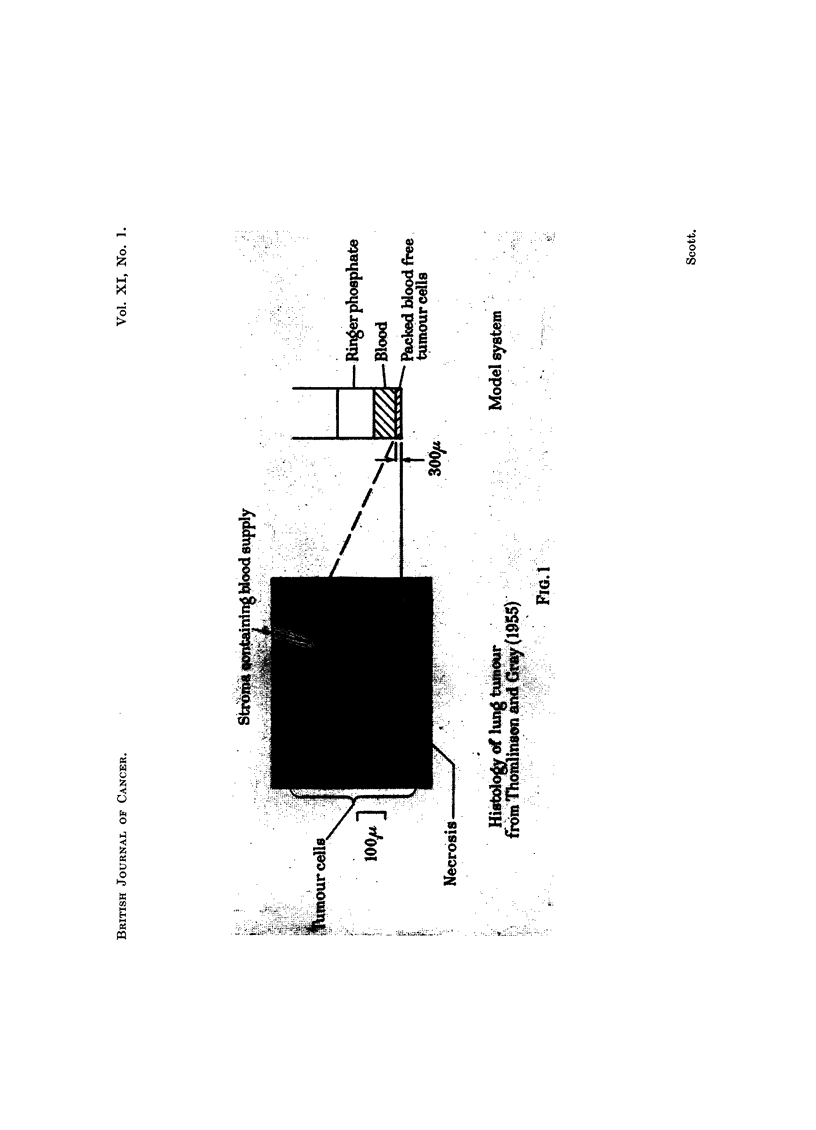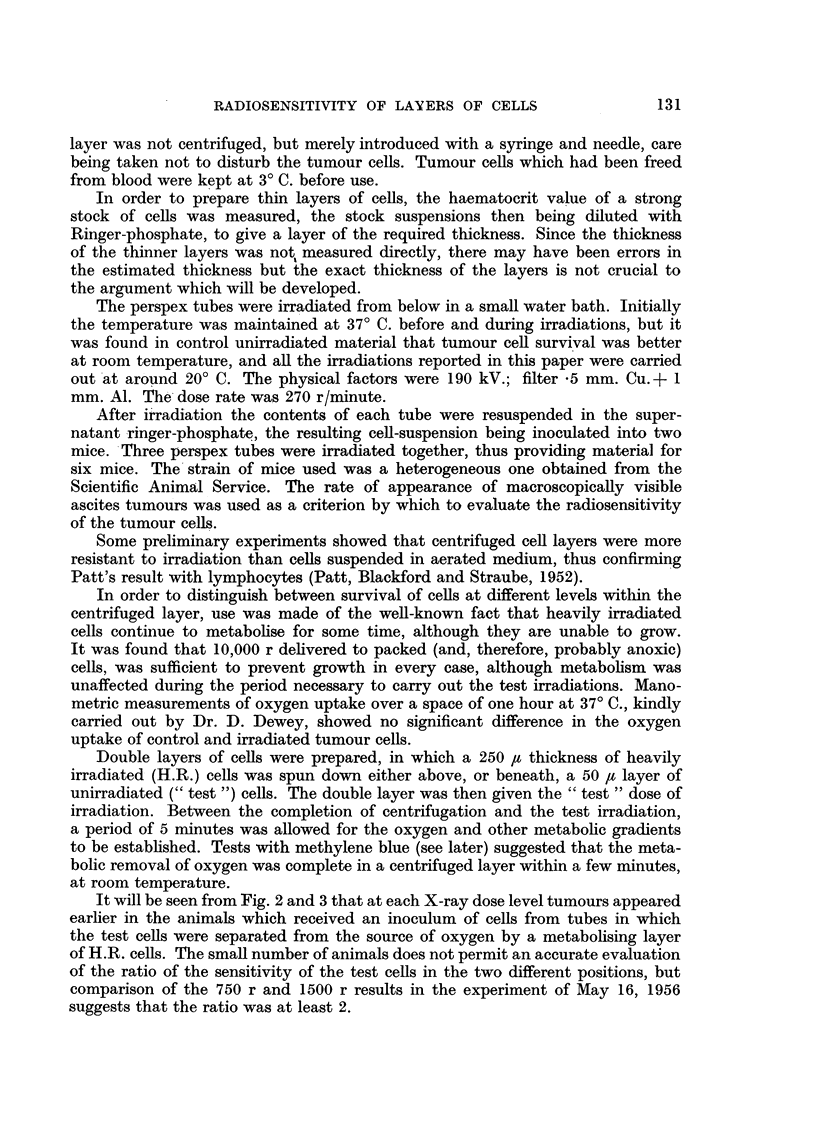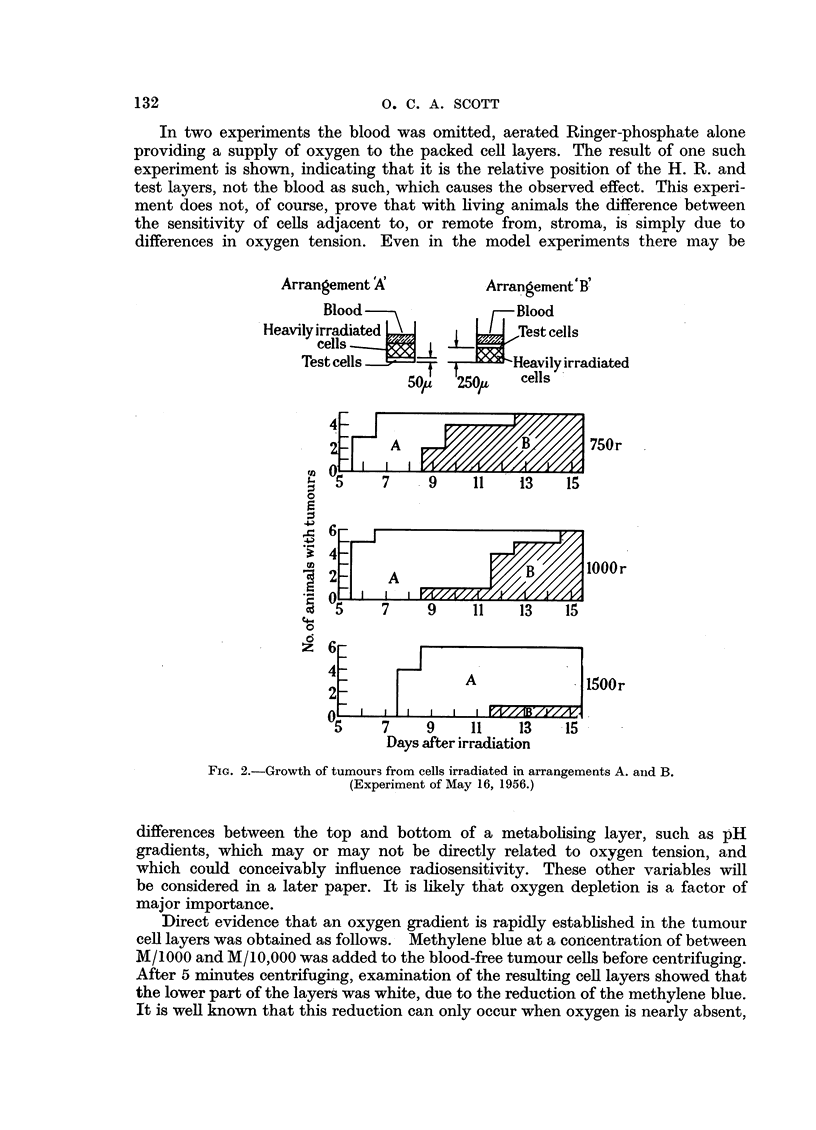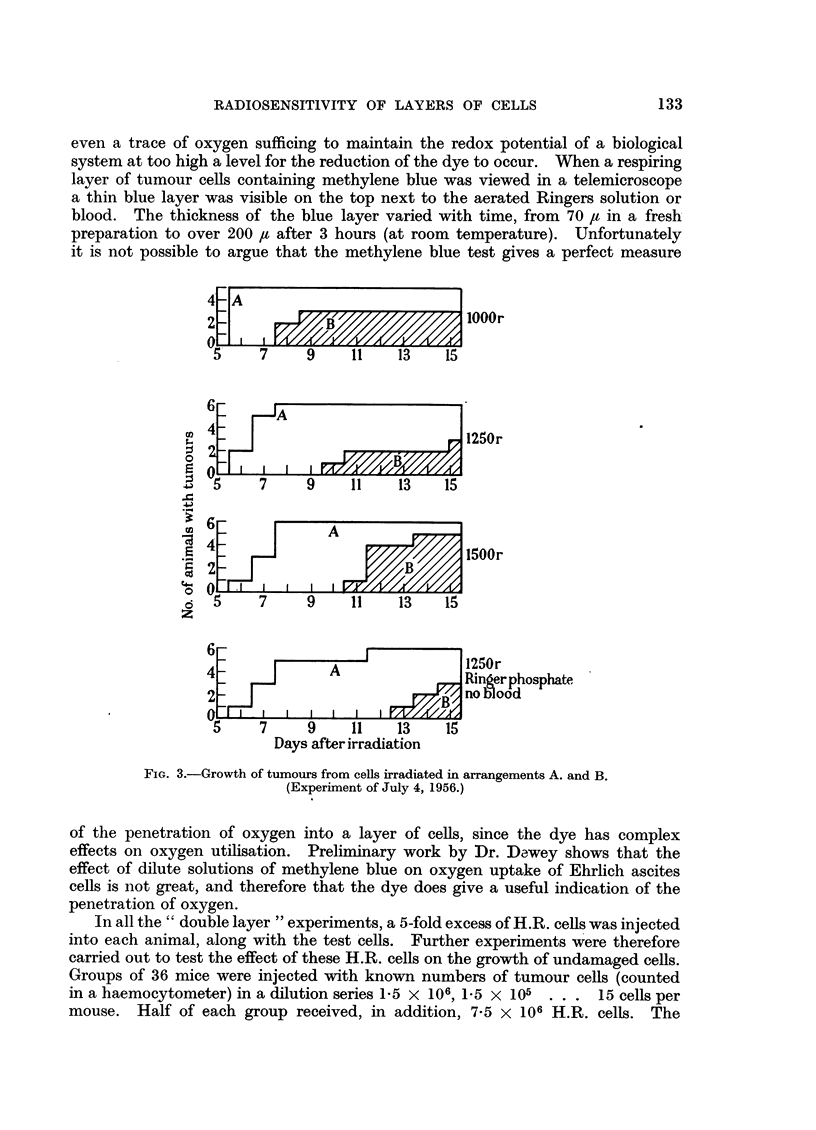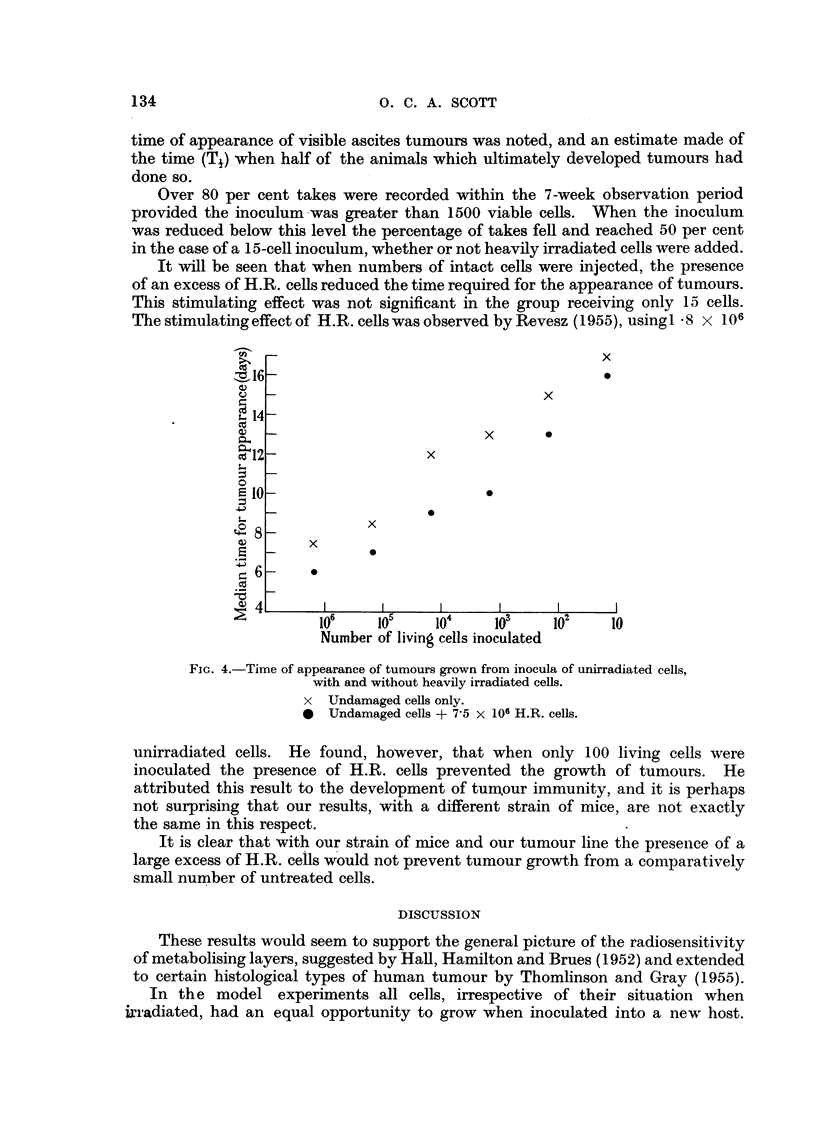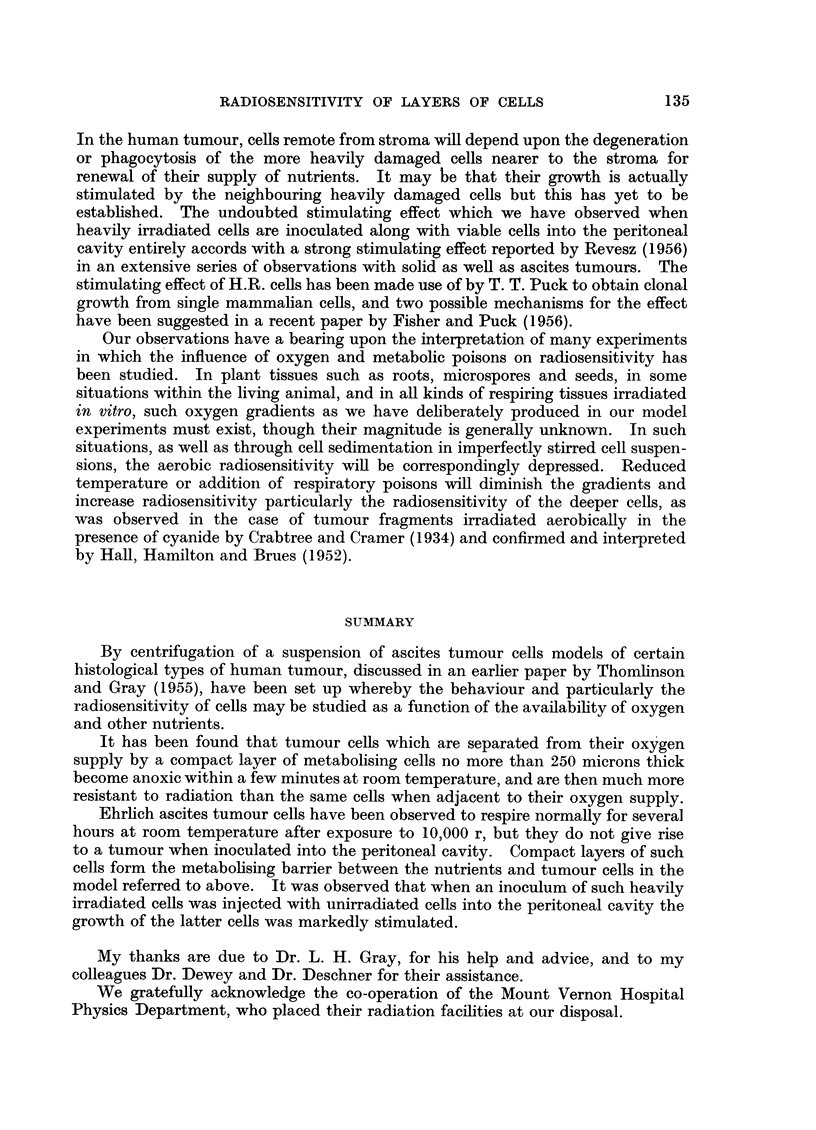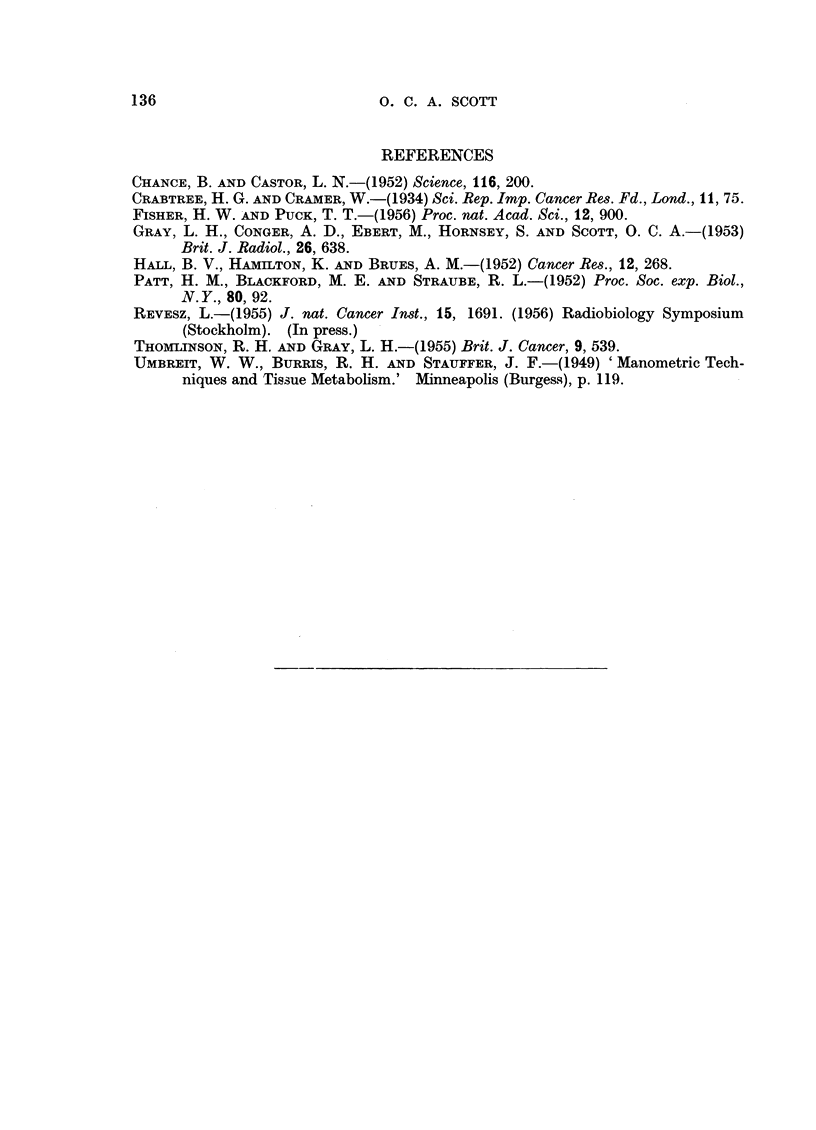# A Model System for Examining the Radiosensitivity of Metabolising Layers of Cells

**DOI:** 10.1038/bjc.1957.19

**Published:** 1957-03

**Authors:** O. C. A. Scott

## Abstract

**Images:**


### A MODEL SYSTEM FOR EXAMINING THE RADIOSENSITIVITY OF METABOLISING LAYERS OF CELLS

O. C. A. SCOTT

*From the Research Unit in Radiobiology, Mount Vernon Hospital and the Radium Institute, Northwood, Middlesex*

Received for publication November 10, 1956

THOMLINSON AND GRAY (1955) examined the histological structure of some malignant human tumours, and they observed that in some squamous cell carcinomas the tumour cells lie packed close together, without any capillaries lying between them.

They suggested that :—

1. Some of these cells can get their supply of oxygen and metabolites only by diffusion, over distances which may be as great as 170 $\mu$.
2. There must exist a steep oxygen gradient through such masses of metabolising cells.
3. Cells located far from the oxygen supply in the stroma may be relatively resistant to damage by X-rays. (Gray, Conger, Ebert, Hornsey and Scott, 1953.)

Direct proof of the survival of individual cells and their descendants from such partially, or wholly, anoxic regions is difficult to obtain by purely histological methods. It was, therefore, decided to construct a model system, in which it would be possible to identify unambiguously the cells which survived a dose of X-irradiation.

To simulate the dense areas of packed cells observed by Thomlinson and Gray (1955), tumour cells from the Ehrlich ascites tumour were centrifuged in flat-bottomed perspex tubes of 1·5 cm. internal diameter. It was found that reasonably even layers of cells could be deposited. The oxygen supply from the stroma of a tumour was simulated by placing a layer of oxygenated (human) blood on top of the tumour cells. The Ehrlich tumour cells are frequently contaminated by blood, and this was removed by the osmotic shock method described by Chance and Castor (1952) (30 seconds in an excess of distilled water followed by addition of an equal volume of double strength saline). The cells were then separated by centrifugation and resuspended in Krebs Ringer-phosphate (pH 7·2) (Umbrett, Burris and Stauffer, 1949). The red blood cells were removed from the tumour cells, because the presence of haemoglobin among the tumour cells in the packed layers would have increased the time required to reach the equilibrium condition, when the amount of oxygen entering the layer is equal to that consumed. The human blood which provided the oxygen supply for the

EXPLANATION OF PLATE.

FIG. 1.—Comparison between model system and histological section human carcinoma of bronchus.

Stroma containing blood supply

Tumour cells

100μ

Necrosis

300μ

Ringer phosphate

Blood

Packed blood free tumour cells

Histology of lung tumour from Thomlinson and Gray (1955)

Model system

FIG. 1

Scott.

layer was not centrifuged, but merely introduced with a syringe and needle, care being taken not to disturb the tumour cells. Tumour cells which had been freed from blood were kept at 3° C. before use.

In order to prepare thin layers of cells, the haematocrit value of a strong stock of cells was measured, the stock suspensions then being diluted with Ringer-phosphate, to give a layer of the required thickness. Since the thickness of the thinner layers was not measured directly, there may have been errors in the estimated thickness but the exact thickness of the layers is not crucial to the argument which will be developed.

The perspex tubes were irradiated from below in a small water bath. Initially the temperature was maintained at 37° C. before and during irradiations, but it was found in control unirradiated material that tumour cell survival was better at room temperature, and all the irradiations reported in this paper were carried out at around 20° C. The physical factors were 190 kV.; filter ·5 mm. Cu. + 1 mm. Al. The dose rate was 270 r/minute.

After irradiation the contents of each tube were resuspended in the supernatant ringer-phosphate, the resulting cell-suspension being inoculated into two mice. Three perspex tubes were irradiated together, thus providing material for six mice. The strain of mice used was a heterogeneous one obtained from the Scientific Animal Service. The rate of appearance of macroscopically visible ascites tumours was used as a criterion by which to evaluate the radiosensitivity of the tumour cells.

Some preliminary experiments showed that centrifuged cell layers were more resistant to irradiation than cells suspended in aerated medium, thus confirming Patt's result with lymphocytes (Patt, Blackford and Straube, 1952).

In order to distinguish between survival of cells at different levels within the centrifuged layer, use was made of the well-known fact that heavily irradiated cells continue to metabolise for some time, although they are unable to grow. It was found that 10,000 r delivered to packed (and, therefore, probably anoxic) cells, was sufficient to prevent growth in every case, although metabolism was unaffected during the period necessary to carry out the test irradiations. Manometric measurements of oxygen uptake over a space of one hour at 37° C., kindly carried out by Dr. D. Dewey, showed no significant difference in the oxygen uptake of control and irradiated tumour cells.

Double layers of cells were prepared, in which a 250 $\mu$ thickness of heavily irradiated (H.R.) cells was spun down either above, or beneath, a 50 $\mu$ layer of unirradiated (" test ") cells. The double layer was then given the " test " dose of irradiation. Between the completion of centrifugation and the test irradiation, a period of 5 minutes was allowed for the oxygen and other metabolic gradients to be established. Tests with methylene blue (see later) suggested that the metabolic removal of oxygen was complete in a centrifuged layer within a few minutes, at room temperature.

It will be seen from Fig. 2 and 3 that at each X-ray dose level tumours appeared earlier in the animals which received an inoculum of cells from tubes in which the test cells were separated from the source of oxygen by a metabolising layer of H.R. cells. The small number of animals does not permit an accurate evaluation of the ratio of the sensitivity of the test cells in the two different positions, but comparison of the 750 r and 1500 r results in the experiment of May 16, 1956 suggests that the ratio was at least 2.

In two experiments the blood was omitted, aerated Ringer-phosphate alone providing a supply of oxygen to the packed cell layers. The result of one such experiment is shown, indicating that it is the relative position of the H. R. and test layers, not the blood as such, which causes the observed effect. This experiment does not, of course, prove that with living animals the difference between the sensitivity of cells adjacent to, or remote from, stroma, is simply due to differences in oxygen tension. Even in the model experiments there may be

FIG. 2.—Growth of tumours from cells irradiated in arrangements A. and B. (Experiment of May 16, 1956.)

differences between the top and bottom of a metabolising layer, such as pH gradients, which may or may not be directly related to oxygen tension, and which could conceivably influence radiosensitivity. These other variables will be considered in a later paper. It is likely that oxygen depletion is a factor of major importance.

Direct evidence that an oxygen gradient is rapidly established in the tumour cell layers was obtained as follows. Methylene blue at a concentration of between M/1000 and M/10,000 was added to the blood-free tumour cells before centrifuging. After 5 minutes centrifuging, examination of the resulting cell layers showed that the lower part of the layers was white, due to the reduction of the methylene blue. It is well known that this reduction can only occur when oxygen is nearly absent, even a trace of oxygen sufficing to maintain the redox potential of a biological system at too high a level for the reduction of the dye to occur. When a respiring layer of tumour cells containing methylene blue was viewed in a telemicroscope a thin blue layer was visible on the top next to the aerated Ringers solution or blood. The thickness of the blue layer varied with time, from 70 μ in a fresh preparation to over 200 μ after 3 hours (at room temperature). Unfortunately it is not possible to argue that the methylene blue test gives a perfect measure

FIG. 3.—Growth of tumours from cells irradiated in arrangements A. and B. (Experiment of July 4, 1956.)

of the penetration of oxygen into a layer of cells, since the dye has complex effects on oxygen utilisation. Preliminary work by Dr. Dewey shows that the effect of dilute solutions of methylene blue on oxygen uptake of Ehrlich ascites cells is not great, and therefore that the dye does give a useful indication of the penetration of oxygen.

In all the " double layer " experiments, a 5-fold excess of H.R. cells was injected into each animal, along with the test cells. Further experiments were therefore carried out to test the effect of these H.R. cells on the growth of undamaged cells. Groups of 36 mice were injected with known numbers of tumour cells (counted in a haemocytometer) in a dilution series $1\cdot5 \times 10^6$, $1\cdot5 \times 10^5$ . . . 15 cells per mouse. Half of each group received, in addition, $7\cdot5 \times 10^6$ H.R. cells. The time of appearance of visible ascites tumours was noted, and an estimate made of the time ($T_{\frac{1}{2}}$) when half of the animals which ultimately developed tumours had done so.

Over 80 per cent takes were recorded within the 7-week observation period provided the inoculum was greater than 1500 viable cells. When the inoculum was reduced below this level the percentage of takes fell and reached 50 per cent in the case of a 15-cell inoculum, whether or not heavily irradiated cells were added.

It will be seen that when numbers of intact cells were injected, the presence of an excess of H.R. cells reduced the time required for the appearance of tumours. This stimulating effect was not significant in the group receiving only 15 cells. The stimulating effect of H.R. cells was observed by Revesz (1955), using1 ·8 × $10^6$

FIG. 4.—Time of appearance of tumours grown from inocula of unirradiated cells, with and without heavily irradiated cells.
× Undamaged cells only.
● Undamaged cells + 7·5 × $10^6$ H.R. cells.

unirradiated cells. He found, however, that when only 100 living cells were inoculated the presence of H.R. cells prevented the growth of tumours. He attributed this result to the development of tumour immunity, and it is perhaps not surprising that our results, with a different strain of mice, are not exactly the same in this respect.

It is clear that with our strain of mice and our tumour line the presence of a large excess of H.R. cells would not prevent tumour growth from a comparatively small number of untreated cells.

DISCUSSION

These results would seem to support the general picture of the radiosensitivity of metabolising layers, suggested by Hall, Hamilton and Brues (1952) and extended to certain histological types of human tumour by Thomlinson and Gray (1955). In the model experiments all cells, irrespective of their situation when irradiated, had an equal opportunity to grow when inoculated into a new host.

In the human tumour, cells remote from stroma will depend upon the degeneration or phagocytosis of the more heavily damaged cells nearer to the stroma for renewal of their supply of nutrients. It may be that their growth is actually stimulated by the neighbouring heavily damaged cells but this has yet to be established. The undoubted stimulating effect which we have observed when heavily irradiated cells are inoculated along with viable cells into the peritoneal cavity entirely accords with a strong stimulating effect reported by Revesz (1956) in an extensive series of observations with solid as well as ascites tumours. The stimulating effect of H.R. cells has been made use of by T. T. Puck to obtain clonal growth from single mammalian cells, and two possible mechanisms for the effect have been suggested in a recent paper by Fisher and Puck (1956).

Our observations have a bearing upon the interpretation of many experiments in which the influence of oxygen and metabolic poisons on radiosensitivity has been studied. In plant tissues such as roots, microspores and seeds, in some situations within the living animal, and in all kinds of respiring tissues irradiated *in vitro*, such oxygen gradients as we have deliberately produced in our model experiments must exist, though their magnitude is generally unknown. In such situations, as well as through cell sedimentation in imperfectly stirred cell suspensions, the aerobic radiosensitivity will be correspondingly depressed. Reduced temperature or addition of respiratory poisons will diminish the gradients and increase radiosensitivity particularly the radiosensitivity of the deeper cells, as was observed in the case of tumour fragments irradiated aerobically in the presence of cyanide by Crabtree and Cramer (1934) and confirmed and interpreted by Hall, Hamilton and Brues (1952).

### SUMMARY

By centrifugation of a suspension of ascites tumour cells models of certain histological types of human tumour, discussed in an earlier paper by Thomlinson and Gray (1955), have been set up whereby the behaviour and particularly the radiosensitivity of cells may be studied as a function of the availability of oxygen and other nutrients.

It has been found that tumour cells which are separated from their oxygen supply by a compact layer of metabolising cells no more than 250 microns thick become anoxic within a few minutes at room temperature, and are then much more resistant to radiation than the same cells when adjacent to their oxygen supply.

Ehrlich ascites tumour cells have been observed to respire normally for several hours at room temperature after exposure to 10,000 r, but they do not give rise to a tumour when inoculated into the peritoneal cavity. Compact layers of such cells form the metabolising barrier between the nutrients and tumour cells in the model referred to above. It was observed that when an inoculum of such heavily irradiated cells was injected with unirradiated cells into the peritoneal cavity the growth of the latter cells was markedly stimulated.

My thanks are due to Dr. L. H. Gray, for his help and advice, and to my colleagues Dr. Dewey and Dr. Deschner for their assistance.

We gratefully acknowledge the co-operation of the Mount Vernon Hospital Physics Department, who placed their radiation facilities at our disposal.

### REFERENCES

Chance, B. and Castor, L. N.—(1952) *Science*, **116**, 200.
Crabtree, H. G. and Cramer, W.—(1934) *Sci. Rep. Imp. Cancer Res. Fd., Lond.*, **11**, 75.
Fisher, H. W. and Puck, T. T.—(1956) *Proc. nat. Acad. Sci.*, **12**, 900.
Gray, L. H., Conger, A. D., Ebert, M., Hornsey, S. and Scott, O. C. A.—(1953) *Brit. J. Radiol.*, **26**, 638.
Hall, B. V., Hamilton, K. and Brues, A. M.—(1952) *Cancer Res.*, **12**, 268.
Patt, H. M., Blackford, M. E. and Straube, R. L.—(1952) *Proc. Soc. exp. Biol., N.Y.*, **80**, 92.
Revesz, L.—(1955) *J. nat. Cancer Inst.*, **15**, 1691. (1956) Radiobiology Symposium (Stockholm). (In press.)
Thomlinson, R. H. and Gray, L. H.—(1955) *Brit. J. Cancer*, **9**, 539.
Umbreit, W. W., Burris, R. H. and Stauffer, J. F.—(1949) 'Manometric Techniques and Tissue Metabolism.' Minneapolis (Burgess), p. 119.